# Autonomous modeling of repetitive movement for rehabilitation exercise monitoring

**DOI:** 10.1186/s12911-022-01907-5

**Published:** 2022-07-03

**Authors:** Prayook Jatesiktat, Guan Ming Lim, Christopher Wee Keong Kuah, Dollaporn Anopas, Wei Tech Ang

**Affiliations:** 1 Rehabilitation Research Institute of Singapore, Nanyang Technological University, Singapore, Singapore; 2 School of Mechanical and Aerospace Engineering, Nanyang Technological University, Singapore, Singapore; 3 Rehabilitation Centre, Centre for Advanced Rehabilitation Therapeutics, Tan Tock Seng Hospital, Singapore, Singapore; 4 Biodesign Innovation Center, Department of Parasitology, Faculty of Medicine Siriraj Hospital, Mahidol University, Bangkok, Thailand

**Keywords:** Anomaly detection, Rehabilitation exercise, Repetitive movement, Segmentation, Time normalization, Upper limb kinematics, Exercise monitoring

## Abstract

**Background:**

Insightful feedback generation for daily home-based stroke rehabilitation is currently unavailable due to the inefficiency of exercise inspection done by therapists. We aim to produce a compact anomaly representation that allows a therapist to pay attention to only a few specific sections in a long exercise session record and boost their efficiency in feedback generation.

**Methods:**

This study proposes a data-driven technique to model a repetitive exercise using unsupervised phase learning on an artificial neural network and statistical learning on principal component analysis (PCA). After a model is built on a set of normal healthy movements, the model can be used to extract a sequence of anomaly scores from a movement of the same prescription.

**Results:**

The method not only works on a standard marker-based motion capture system but also performs well on a more compact and affordable motion capture system based-on Kinect V2 and wrist-worn inertial measurement units that can be used at home. An evaluation of four different exercises shows its potential in separating anomalous movements from normal ones with an average area under the curve (AUC) of 0.9872 even on the compact motion capture system.

**Conclusions:**

The proposed processing technique has the potential to help clinicians in providing high-quality feedback for telerehabilitation in a more scalable way.

## Introduction

An increasing number of stroke survivors globally leads to a growing demand for rehabilitation and long-term care [1]. However, the continuity of care at the patient’s home is limited as a therapist cannot conduct time-consuming home visit regularly [2]. Moreover, the home visit could be expensive and not widely available [3]. This lack of monitoring when the patient performs self-directed exercises makes it difficult for the therapist to assess whether the prescribed exercises are done properly. As such, telerehabilitation services that allows the therapist to monitor the exercise session remotely [4] have been developed to enhance functional recovery in stroke patients [5–7]. Considering a typical exercise duration of around 30–60 min per day for each patient [8], a therapist will still be overwhelmed by long hours of information generated daily from all the patients under his supervision. To make the daily feedback generation scalable, an automated data analysis must be integrated on top of a sensing system to reduce the therapist’s workload [9].

To reduce the therapist’s workload, motion capture system with machine learning have been used to automate the assessment of rehabilitation exercise [9, 10]. For example, Raihan’s study employed convolutional neural networks to predict the summary score of rehabilitation exercise sessions [9]. Similarly, various studies use motion-related sensors with data-driven techniques to calculate scores for assessments [11–15]. For instance, Lee’s study used time-frequency informed singular spectrum analysis to automatically assess the score of one grasping task in the Action Research Arm Test (ARAT) [11]. However, these studies can only show a summary score of each rehabilitation session. By looking at those scores, a therapist cannot pinpoint an anomalous movement of patients to generate insightful feedback. Some studies designed the compensation detection methods for patients [16–19]. Nevertheless, methods in these studies have not been designed to produce anomaly detection in a time-series manner.

Interestingly, there is a study [20] that calculates time-series anomaly scores of repetitive tasks involving upper body movements. Their method detects intra-subject anomalies based on a history of movement from the same healthy subject in the earlier part of the same episode. Thus, it is useful for fatigue detection in assembly line workers performing repetitive tasks. On the other hand, our method is designed for monitoring stroke survivors performing rehabilitation exercises. Furthermore, in our case, the movement anomaly cannot be compared against the same patient, but it must be compared against a set of normal healthy movements from a group of healthy subjects.

This study aims to design an information processing method that assists a therapist in monitoring their patients who need daily rehabilitation exercises. By automatically transforming the movement records into a time-series plot of anomaly scores, it can help to direct the therapist’s attention to specific sections of the records where the patient performs the exercises differently. This will allow the therapist to have more time in generating insightful feedback instead of aimlessly skimming through the full length of the video record to identify the anomalies. A similar form of automatic assessment during the rehabilitation exercise has been found in the Virtual Exercise Rehabilitation Assistant (VERA) for lower extremity exercises [21]. However, the feedback generated from VERA must follow pre-defined criteria of correct movement which is programmed individually for each exercise.

Instead of engineering a new set of rules for every new movement prescription, our system is designed to automatically model some characteristics of the normal exercise from a small group of healthy subjects performing the prescription. To do so, there are a number of requirements and challenges as follows.

1. Due to the impracticality of using a multi-camera marker-based motion capture system at a patient’s home on a daily basis, the method must be tolerant of inaccuracy in the data recorded from a more compact motion capture system (e.g., with a single depth camera).
2. A patient undergoing rehabilitation usually moves slower as compared to healthy subjects. The method must not consider those slow movements as anomalies if the movement patterns are similar to the norm.
3. Other than the data collection step from healthy subjects, all the other steps such as period segmentation must be done automatically without any given template of the waveform. The method only has access to records of continuous repetitive movements from healthy subjects in order to model the movement.
4. When a therapist wants to add a new exercise movement, it should not be too time-consuming. Thus, the number of healthy subjects needed to create the new exercise model should not be too large (10–12 subjects). With this small training data, the generalization to an unseen subject is one of the challenges.

To overcome those challenges, the time segmentation and time normalization of our method largely rely on our previous work on unsupervised phase learning and extraction using neural network [22–25]. This technique allows us to effectively use a standard anomaly detection technique such as principal component analysis (PCA) reconstruction error [26]. In addition, the sequence of the extracted phase itself can also be a clue to enhance anomaly detection.

In the literature, the work of Murgia et al. [27] is closest to our requirements. They model a cyclic movement of a page-turning task using mean and standard deviation at each phase of the movement which forms a series of confidence intervals from the beginning to the end of one movement period. If the active range of motion (AROM) falls outside the confident range, compensation or a lack of movement will be highlighted. During the record, the subject needs to push a button to mark the beginning and the end of each period for segmentation and time normalization. Similar digital markers or rule-based techniques for automatic segmentation are commonly found in the analysis of upper limb movements [15, 28]. However, they are designed to model one specific task which does not match our requirements. To make it more general in our study, the phase extraction and the segmentation are done with data-driven techniques instead.

## Method

**Fig. 1:**
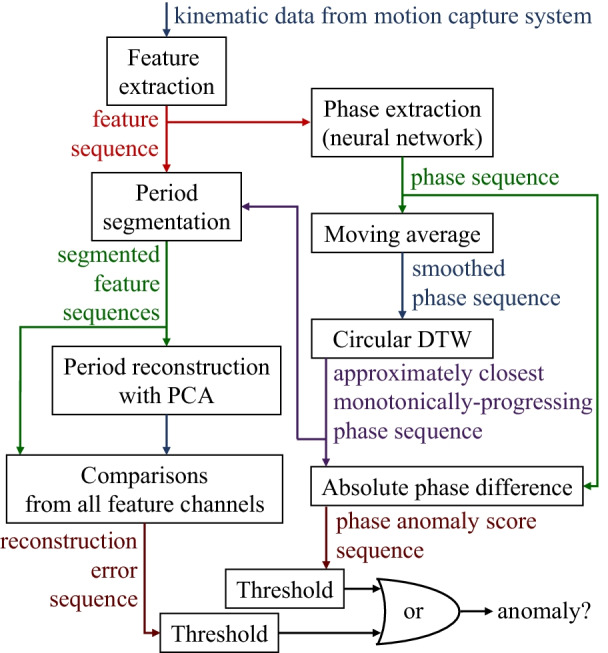
A data flow diagram of all the key steps in anomaly detection. An anomaly can be detected by either an abnormality in a period waveform (flow on the left side) or a fluctuation of the extracted phase (flow on the right side)

The method is composed of two stages. The first stage is the modeling stage in which all the parameters (e.g., neural network weights and biases for phase extraction, dimensionality reduction matrices from principal component analysis, reconstruction-error normalization parameters) will be calculated from records of unsegmented repeated movement from a group of healthy subjects. The second stage is the anomaly detection in the actual exercise sequence which can be seen in Fig. 1.

The method is designed to detect anomalies from two main clues that are 1) the fluctuation in the phase progression and 2) the deviation of kinematic features from the healthy norm.

All the contents in this method section are from the first author’s Ph.D. thesis [29].

### Motion capture systems

The kinematic data of subjects’ movements are collected from three motion capture systems for comparison. The first system is Microsoft Kinect V2 with skeleton tracking capability from Kinect Software Development Kit 2.0 (SDK) [30–32]. The second system is a combination of the Kinect and wrist-worn inertial measurement units with model personalization to enhance the pose tracking accuracy [33]. The third system is a marker-based multi-camera motion capture system (Qualisys) [34]. These three systems with increasing level of tracking accuracy allow us to observe the effect of sensing noise on anomaly detection. For reference purposes, the first, the second, and the third system will be called *Kinect Alone*, *Kinect+IMU*, and *Marker-based* respectively. Each system records data at 30 frames per second.

**Fig. 2:**
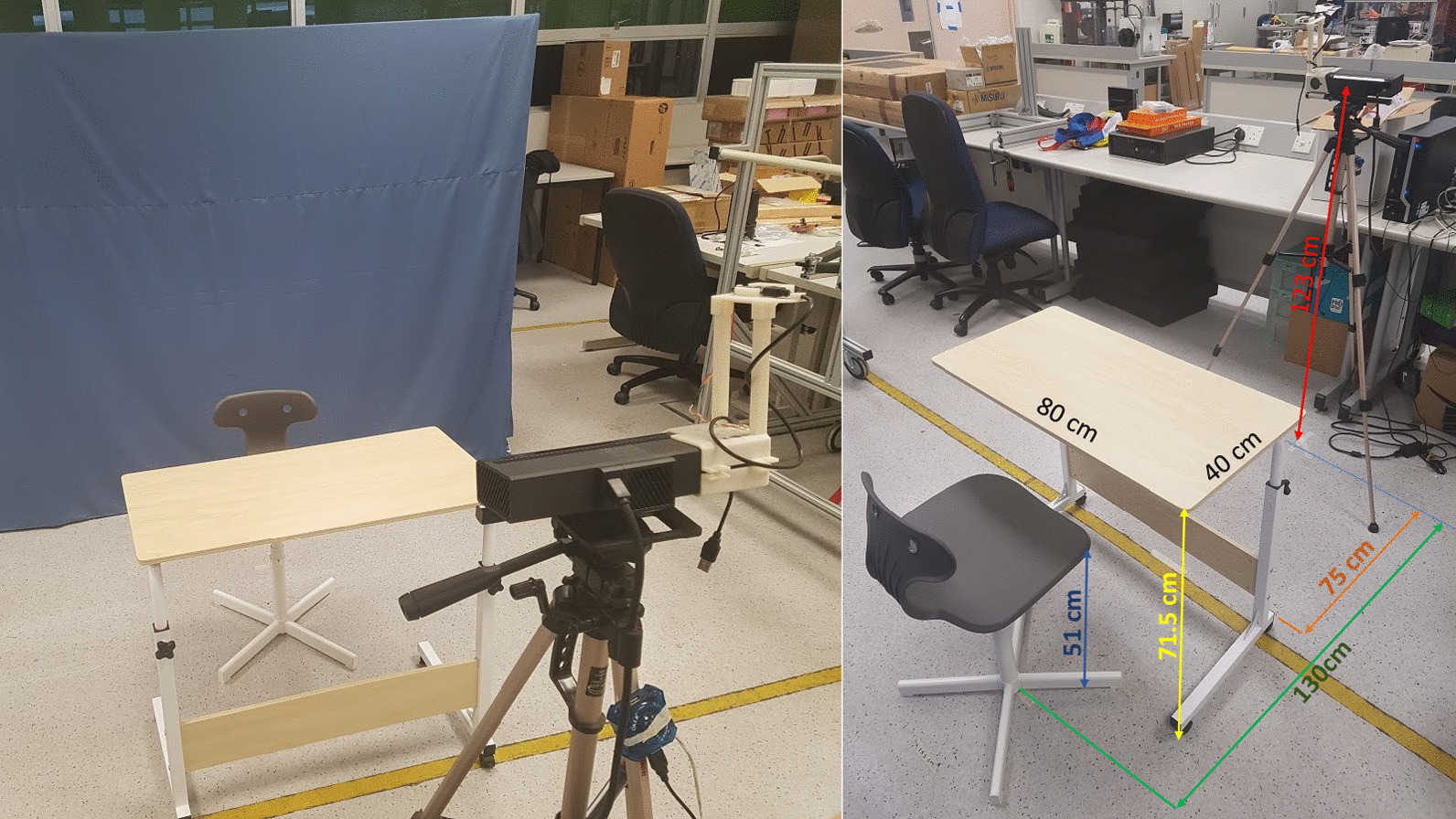
The table, chair, and the camera setup for *Kinect Alone* and *Kinect+IMU* motion capture system. This arrangement is also used in a marker-based motion capture volume to perform simultaneous records from all three motion capture systems

The position of the Kinect camera with the table and the chair arrangements are illustrated in Fig. 2. The records are always done in a brightly lit and quiet room without external distraction.

### Selected features

This study focuses on unilateral exercises of gross upper-limb movements. Therefore, the selected features are limited to the upper torso, the right upper arm, and the right forearm. These features are

- upper-arm pointing direction (3D unit vector) using upper torso as the frame of reference [UA Pnt.],
- forearm pointing direction (3D unit vector) using upper torso as the frame of reference [FA Pnt.],
- upper torso frontal flexion [TS Ffx.],
- upper torso left/right turning angle [TS Turn],
- upper torso lateral flexion [TS Lfx.],
- forearm pronation/supination turning angle [FA Prn.],
- wrist position (X-component) in the *core* reference frame [WR X],
- wrist position (Y-component) in the *core* reference frame [WR Y],
- wrist position (Z-component) in the *core* reference frame [WR Z].

Each bullet is treated as one *feature channel*. The concept of feature channel will play a role in the *Detect Anomaly from Reconstruction Error* section.

The *core* reference frame is a static frame for each record defined at the first frame of recording when the subject is at a normal resting posture. The origin of *core* reference frame is defined as the middle point between two shoulder locations that is shifted downward (along the gravity direction) to the table level. The Y-axis of the *core* frame points in the upward direction (opposite to downward gravity). The Z-axis of the *core* frame points from the origin parallel to the ground towards the camera. Then, the X-axis of the *core* frame can be calculated from Y-axis and Z-axis using a cross product.

For the forearm pronation, the zero-degree angle is defined by the pose that the thumb pointing direction is parallel to the plane perpendicular to the folding axis of the elbow. More pronation from that reference angle will give a positive value and more supination from that reference angle will give a negative value.

For the upper torso turning angle, the zero-degree angle is defined by the pose of the upper torso that is facing toward the camera. Turning to the left causes the value to be more positive.

For the upper torso frontal tilt and lateral tilt, they are calculated using vertical direction from gravity as the reference. A positive angle for the frontal tilt means tilting forward. A positive angle for lateral tilt means tilting to the right side.

### Azimuthal equidistant (AZEQ) projection

For the first two features, the data are distributed on a unit sphere. This curvy representation is problematic for our data reconstruction process. For example, linear reconstruction from principal component analysis (PCA) of spherical data will not stay spherical. Therefore, a new representation is proposed to ensure the validity of the reconstruction. At the same time, it must avoid the discontinuity of the data at the potential wrapping point to facilitate the learning process.

**Fig. 3:**
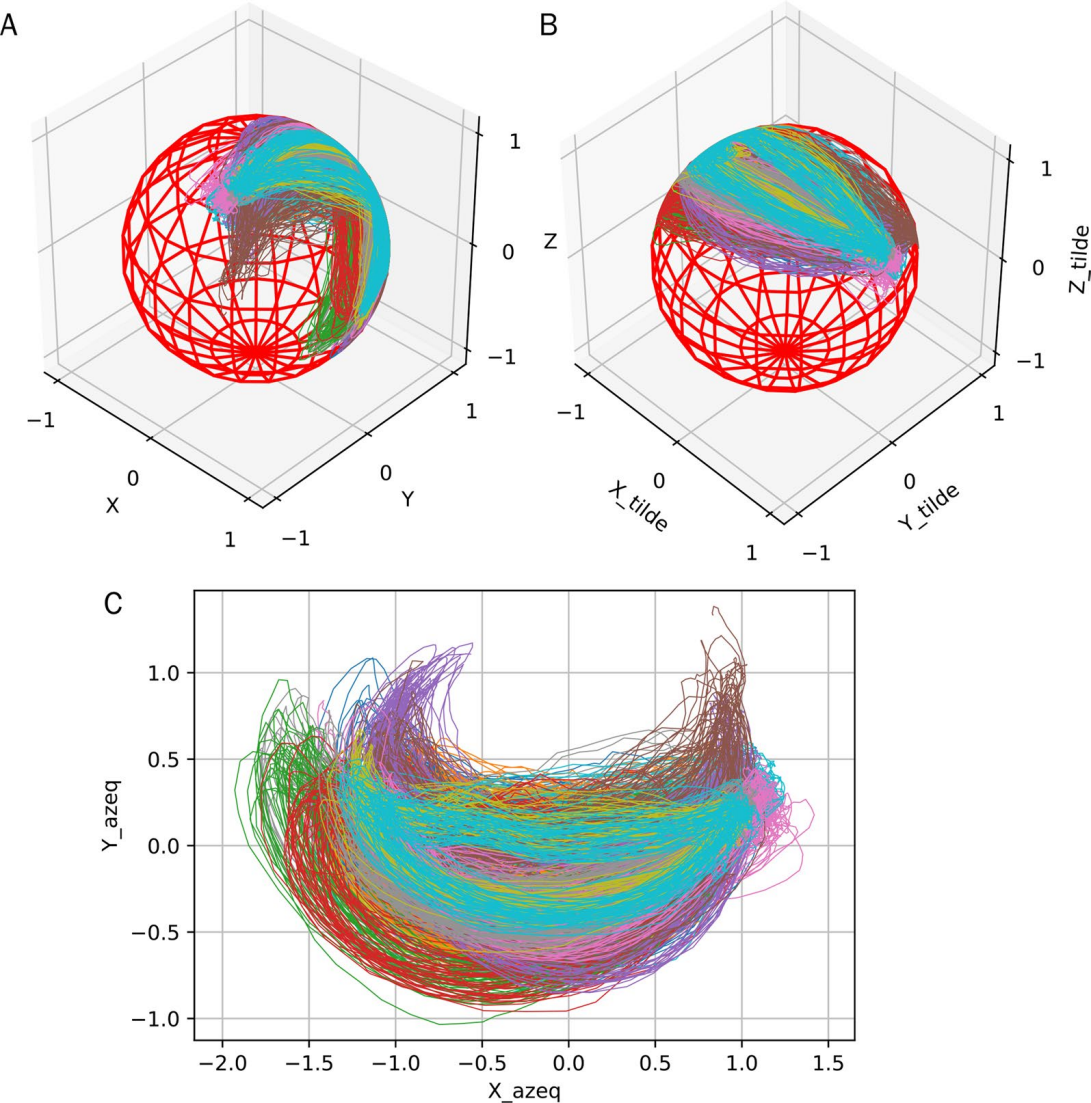
Trajectory of forearm pointing direction of COMB movement from multiple subjects. **a** Original 3D representation in the upper-torso reference frame. **b** Rotated to be around the north pole. **c** Result of azimuthal equidistant projection

The solution as illustrated in Fig. 3 is to first rotate the spherical training data around the origin to make the mean of the training data stay on the positive side of the Z-axis (the top part of the sphere). Then, the azimuthal equidistant projection is applied to transform the data into a flat 2D space. By this projection, the point at the north pole will be at the origin of the new 2D space. The point at the south pole will be torn and stretched out to become a circle with a radius of $\pi$ in the new 2D space. Since the warping point is at the south pole, the pre-rotation of the training data to the north pole is necessary to minimise the chance of the data getting close the south pole.

This projection converts the 3-dimensional upper-arm pointing direction and the 3-dimensional forearm pointing direction into a form of a 2-dimensional vector which can be reverted into its original form. The conversion of each channel is parameterized by a 3-by-3 rotation matrix calculated from the training data.

### Unsupervised phase learning and extraction

The next step is to extract a sequence of phases from the multi-dimensional time-series data of pose features. A learning method from our previous work [23] is used to learn a proper way to transform a small time-window of data of all feature channels into a phase value. The training is done in an unsupervised manner without the need for phase labels or pre-segmentation in the training data. The unlabeled data just need to be quasi-periodic time-series data and it can be multi-dimensional. The internal architecture of this learning method is multiple layers of fully-connected neural networks but the cost function used to train this network is specially designed to learn from the relation of every two consecutive signal windows in the training dataset. After the training, the weights and biases of the neural network will be used for phase extraction via a standard feed-forwarding method. The network outputs a phase represented by a point on a unit circle (*x*, *y*). It can be converted into 1D representation of phase by $\phi =\arctan 2(y,x)\%(2\pi )$. Since the $\arctan 2$ operator has a range of $(-\pi ,\pi ]$, the modulo operator (%) is used to turn it to a positive range of $[0,2\pi )$.

The output from the phase extraction is necessary for period segmentation and anomaly detection from phase fluctuation.

### Phase sequence smoothing

Sometimes, noise and error from the pose extraction step could lead to an unreasonable phase warp. To mitigate this problem, the series of extracted phases (2D representation) will be smoothed by a simple moving average filter followed by a normalization to adjust it back on the unit circle.

### Monotonically progressing phase assignment

By observation, the series of phases extracted from healthy movement always progress in the forward direction (i.e., counter-clockwise direction on a unit circle). A minor exception occurs only when the movement pauses. A pause can generate a tiny fluctuation around the same spot in the extracted phase sequence.

This observation leads to a key assumption, “if a series of phases in a period of movement is deviating from the closest monotonically progressing series, the source movement is also deviating from the normal movement”. According to the assumption, we need to find the closest monotonically progressing series and compare them to the extracted phase. Notice that the phrase *monotonically progressing* is used instead of *monotonically increasing* to agree with the wrapping nature of the phase. If a phase that almost reaches $2\pi$ progresses forward, it will wrap around and start from zero again.

In order to search for the exactly closest monotonically increasing series, it requires too much computational power and slows down the process. Therefore, an approximation method is developed by limiting the value of the series to be discrete and evenly spread from 0 to $2\pi$.

This problem can be solved by our proposed variant of the Dynamic Time Warping (DTW) algorithm called *Circular Dynamic Time Warping (CDTW)*. The original DTW [35] is designed to compare two finite sequences that have similar waveforms from the beginning to the end but may have different time-warping structures. It assumes both finite sequences to have the same beginning (at 0% progress) and the same ending (at 100% progress).

In our situation, there are two sequences to compare. The first sequence is the unsegmented smoothed phase sequence. This sequence can start and end at any phase value (from 0 to $2\pi$). In between, it is likely to have continuous phase progression (ramps up to $2\pi$, wraps to 0, and keeps repeating) or some fluctuated phase progressions in the case of an anomaly. The number of rounds progressing in this sequence is unknown. The second sequence (template sequence), in the case of the original DTW, should be a monotonically progressing sequence that starts and ends with the same phase as the first sequence, and it should contain the same number of progressing rounds in between to match perfectly with the first sequence. However, in the case of CDTW, because the number of repetitions is unknown, the second sequence is replaced with a single period from 0 to $2\pi$ and the sequence is modified to have a wrapping point from $2\pi$ to 0.

**Fig. 4:**
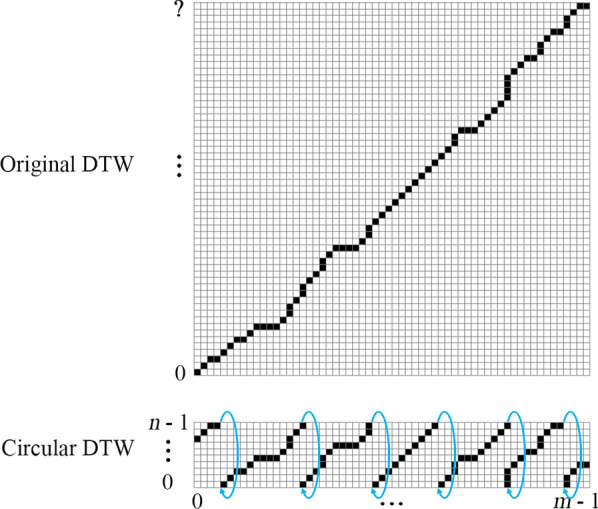
Comparison of the data structure used in the original DTW and Circular DTW. Each black square represents a matching point between one sample in a template sequence and one sample in an unsegmented smoothed phase sequence (output of Algorithm 2). The original DTW is impractical as the proper number of rows is unknown before the calculation. The Circular DTW lets one finite template sequence be reused for multiple rounds as needed by bridging the first and the last row of the table


This modification changes the structure of the minimum distance table in the original DTW algorithm from a rectangle to a cylindrical tube. The length of the first sequence becomes the length of the tube. The length of the template sequence becomes the circumference of the tube. In every round of progress made in the first sequence, the warping result will go around the tube once. Figure 4 illustrates the differences between the table of DTW and CDTW. Algorithm 1 describes how the table of CDTW is calculated given that the distance function between two-phase values is defined as

1 $$\begin{aligned} D(\phi _a,\phi _b)= {\left\{ \begin{array}{ll} \begin{aligned} &{}|(\phi _a-\phi _b)\%(2\pi )|, &{}&{}\text {if } (\phi _a-\phi _b)\%(2\pi ) \le \pi \\ &{}|(\phi _a-\phi _b)\%(2\pi )-2\pi |, &{}&{}\text {otherwise} \end{aligned} \end{array}\right. } \end{aligned}$$

and $circularTemplate[i]=2\pi i/n$ is the template sequence for our application. This sequence is used to associate the index with its phase value.

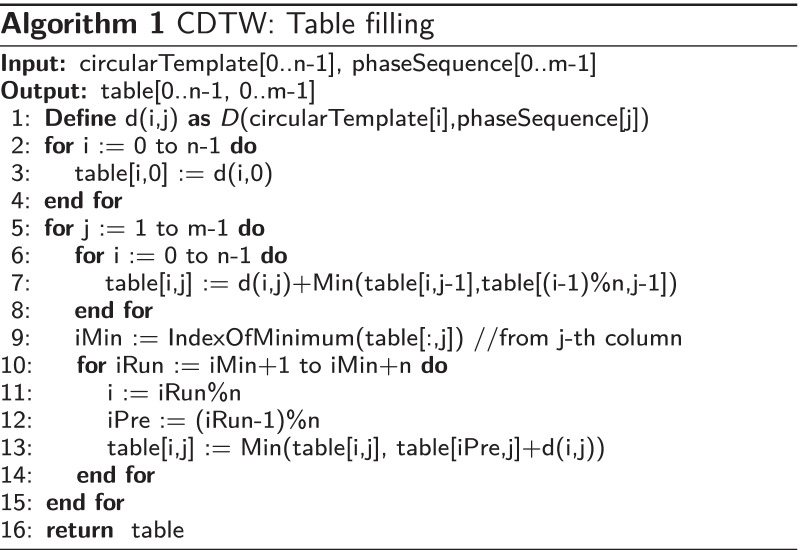

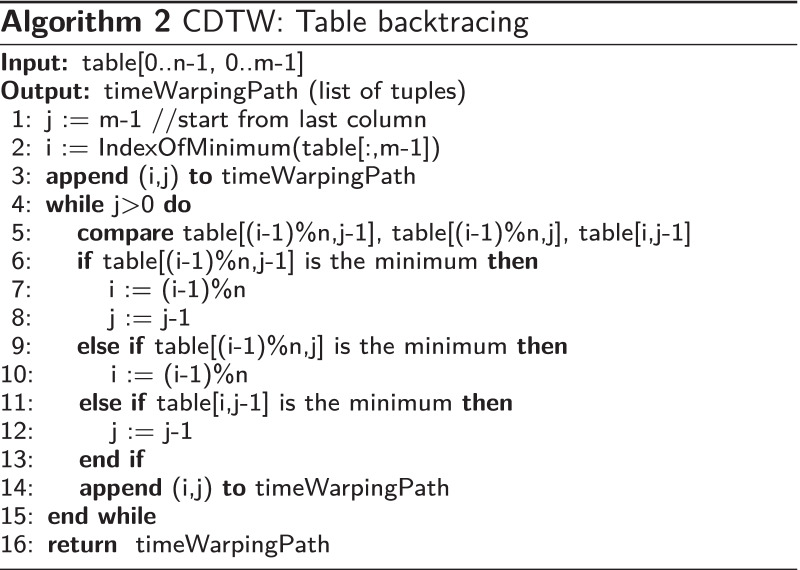


**Fig. 5:**
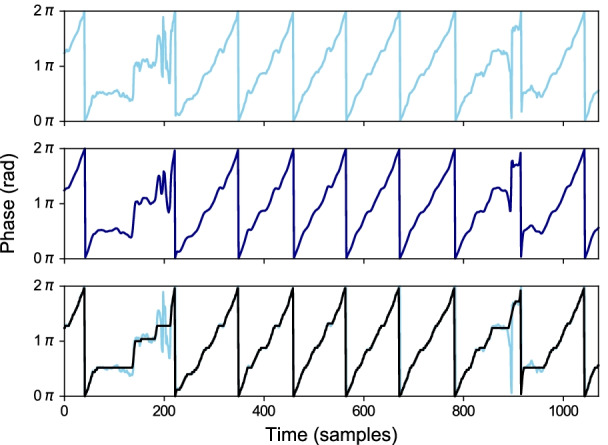
Top: An extracted phase sequence. Middle: Smoothed phase sequence. Bottom: Approximately closest monotonically progressing (ACMP) phase sequence (black) overlaid on top of the extracted phase sequence (light blue). The differences between them are obvious when the extracted phase fluctuates

After the table is calculated, Algorithm 2 is used to trace back from the last column to the first column to find how the time is warped throughout the smoothed phase sequence. From the *timeWarpingPath* output, any specific column *j* will be matched with one or more consecutive rows. Given that $i_{mid}$ is the index at the middle of those consecutive rows, the approximately closest monotonically progressing (ACMP) phase assignment at the *j*th sample is $2\pi i_{mid}/n$. An example of this assignment can be seen in Fig. 5.

In terms of computational performance, CDTW is similar to DTW with the time complexity of $O(m*n)$ given that *n* is the length of the circular template (*n* = 50 in this implementation) and *m* is the length of the extracted phase sequence.

### Detect anomaly from phase progression

The approximately closest monotonically progressing phase sequence is compared to the original extracted phase sequence using Equation (1). The magnitude of the resulting sequence is used to infer anomaly in phase progression.

### Period segmentation

Every point in the approximately closest monotonically progressing (ACMP) phase sequence with the value crosses $2\pi$ to a new round is considered as a separator between two consecutive periods. This segmentation breaks a long continuous exercise sequence into multiple sequences of unequal length. Each segmented sequence is expected to contain one round of exercise.

### Detect anomaly from reconstruction error

**Fig. 6:**
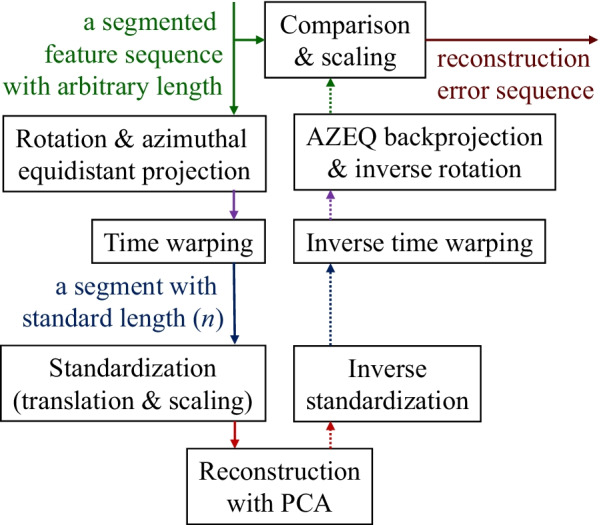
A data flow diagram of reconstruction error calculation. The process is done independently in each feature channel. The rotation and azimuthal equidistant projection are only applicable for the upper arm and forearm pointing direction feature channels

The idea of this section is to reduce the dimension of a segmented period to a lower-dimensional space using PCA and reconstruct it back to the original form of the data to be compared for the error. However, there are a few processing steps before and after the PCA reconstruction as illustrated in Fig. 6.

#### Azimuthal equidistant projection

The process previously described in *Azimuthal Equidistant (AZEQ) Projection* section will be applied to the upper arm and forearm pointing direction feature channels to reduce the number of dimensions per sample to two. All other feature channels will be unaffected and they will have one dimension per sample.

#### Time warping

Because the length of one segment can be arbitrary, each segmented period has to be warped into one standard length of *n* samples to allow the use of PCA reconstruction. The time-warping result from CDTW is used to determine how the segment is warped. If multiple consecutive samples from the original sequence are warped to the same slot in the standard length, the data will be averaged.

#### Period standardization

After the time warping, each feature channel can be seen as a high-dimensional vector with a constant number of dimensions. For the forearm pointing direction and upper-arm pointing direction feature channels, each channel becomes a 2*n*-dimensional vector. For other feature channels, each channel becomes an *n*-dimensional vector.

If the training data are processed until this point, it will produce a collection of such a vector in each feature channel. They are used to calculate standardization parameters (a vector of means and a vector of standard deviation) for each feature channel independently. These standardization parameters are used to translate and scale the data distribution in all the dimensions to have zero mean and unit variance.

#### Principal component analysis (PCA) reconstruction

In the training stage, a dimensionality reduction matrix ($U_{r}$) will be created independently per feature channel to be used in the inference stage. Given that a standardized period from one feature channel is flattened to a $N_c$-dimensional vector (with $N_c=2n$ or *n* depending on the channel), a collection of such vectors from the training data will be fed to the standard PCA to extract all the $N_c$ principal components. The smallest subset of the principal components that explains at least 95% of the variance in the training data will be used to form a dimensionality reduction matrix ($U_{r}$) for that channel. If $N_d$ components are selected, the matrix $U_{r}$ will be $N_d$-by-$N_c$ in size.

During the inference stage, let $\mathbf {c}$ be a flattened $N_c$-dimensional vector from a feature channel of a period and let $U_{r}$ be a dimensionality reduction matrix of the channel. The reconstruction of the vector $\mathbf {c}$ can be calculated by

2 $$\begin{aligned} \hat{\mathbf {c}} = U_{r}^\top U_{r} \mathbf {c} \end{aligned}$$

From right to left, the first matrix multiplication is done to project a vector to a lower-dimensional space. Then, the second multiplication is to reconstruct it back to $N_c$ dimension.

This process acts as a dimensionality bottleneck that forces a group of correlated dimensions to share a single dimension to represent them. If a new unseen period is similar to the period in the training data the reconstruction should preserve the original data quite well. On the other hand, if the period contains anomalies or an incorrect movement that disagrees with the direction of the selected variances in the training data, the reconstruction will differ largely from the input.

#### Reconstruction error

**Fig. 7:**
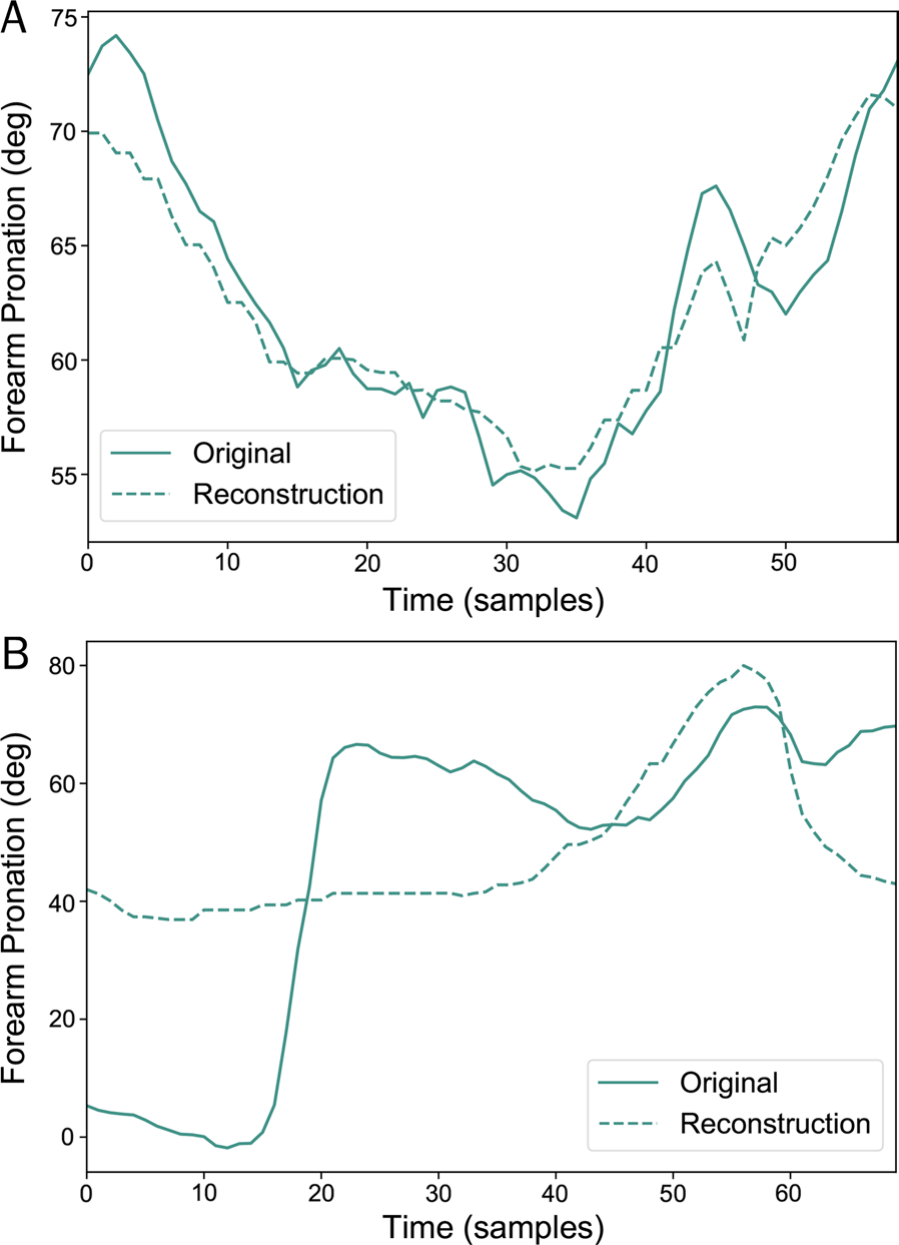
Reconstruction of forearm pronation channel from a segmented period of RECT movement. **a** For a normal movement, the reconstruction will be similar to the recorded data. **b** When an anomaly occurs, the reconstruction does not align well with the original data

According to the upward flow on the right side of Fig. 6, the reconstruction from PCA will be inverted all the way back to the original form and length to be compared to the original segmented sequence sample-to-sample as illustrated in Fig. 7.

For the first two feature channels (upper-arm and forearm pointing direction), the reconstruction error is measured by the angle between two 3D unit vectors (the original and the reconstruction). For other feature channels, the reconstruction error is the element-wise absolute difference. By this calculation, every sample will contain one reconstruction error per feature channel. However, the scales of error of each feature channel at different phases of the movement are largely different. In order to use one threshold for all the reconstruction error channels, they have to be scaled to a similar range.

The time-warping results from the training data can be used to associate a sample to one of the *n* discrete phases. By this method, the statistics of the reconstruction error of a feature channel around a specific phase can be calculated from the training data. We choose the mean square of the reconstruction error as the scaling parameter to adjust all the reconstruction errors to a similar range before applying one global threshold.

## Evaluation

The evaluation is done on two independent experiments. For the first experiment, a model is built from normal movements from healthy subjects (training group), and it is tested on a mixture of normal movements and simulated anomalies done by healthy subjects outside the training group. This experiment is done to quantitatively evaluate the anomaly detection method because it is possible to manually label those simulated anomalies. For the second experiment, a model is built from normal movements from healthy subjects, and it is tested on movements done by post-stroke subjects. As there is no standardized method to label anomalies in their movements, the results from this experiment will be presented in a qualitative manner.

All the evaluation methods in this section are from the first author’s Ph.D. thesis [29].

### Data collection

The data collection involves four repetitive exercises of right arm movements which are **1)** rectangular drawing (RECT), **2)** tap in loop (TAP), **3)** hair combing (COMB), and **4)** water pouring (POUR). All exercises are done in a sitting posture with a table and a Kinect V2 in front of the subject (Fig. 2). For RECT, a subject slides his right hand on a table to draw a rectangle. For TAB, a subject starts with his right hand on a table and raises the hand to touch his nose and left shoulder and puts the hand back on the table. For COMB, a subject starts with his hand on a table and lifts the hand to comb his hair and puts the hand back on the table. For POUR, a subject holds an empty cup and performs a pouring action into a bucket on the table and moves the hand back to the starting position.

For each exercise, the data collection is composed of three groups.

#### Normal movements

Twelve healthy subjects (5 males, 7 females, 25–59 years old) are recruited to perform 100 repetitions per exercise per subject. All subjects are asked to mix slow, medium, and fast paces to their movements for data variations. Those variations are mixed in the record without any label. The speeds of the movements across multiple subjects do not need to be controlled to be the same. This group of data is used to model the norm of each exercise.

#### Simulated mixture of normal and abnormal movements

Each subject in the same group of 12 healthy subjects also performed 40 normal repetitions with insertions of 6 additional repetitions that contain simulated anomalies. Examples of those simulated anomalies are jerks, shaking, overshooting, undershooting, limited range of motion, torso compensation, and unnatural joint coordination. Each anomalous event is labeled by marking the beginning and the end of the event for evaluation purposes. This group of data is used to quantitatively evaluate the method.

#### Movements from post-stroke subjects

Fourteen stroke survivors (6 males, 8 females, 44–86 years old) with their right arm affected are recruited to perform those four exercises. All subjects, except one, are over six months post-stroke. Their Fugl–Meyer upper extremity motor sub-scores for shoulder–elbow-coordination portion range from 19 to 41 with the highest total score of 42 (without wrist and hand portions). This group of data is used to qualitatively evaluate the method. Each movement sequence is simultaneously recorded by three motion capture systems stated in *Motion Capture Systems* section.

### Method validation

#### Experiment on simulated anomalies

To test the generalization capability for the subject outside the training data, the 6-fold cross-validation is used. In each round of validation, the normal movements from 10 subjects will be used to build a model, and the mixture of normal and abnormal movements from the other two subjects will be used to test the model. The evaluation is repeated 6 times with different partitioning to cover all subjects in the testing step.

The testing output in a record will initially appear in two forms in any sample. The first one is the anomaly score from phase progression (1 dimension). One threshold is needed to flag anomalies from this measure. The second one is the anomaly score from feature reconstruction errors (9 dimensions) from 9 feature channels. Since this measure is already scaled to have the same range, one threshold will be used against all dimensions. The absolute value of all scaled reconstruction errors will be compared against this threshold to flag anomaly. If at least one sample in an anomaly event is flagged, that anomaly will be counted as detected (true positive). If at least one sample in a normal period is flagged, the period will be counted as a false anomaly (false positive).

To evaluate the capability of these proposed measures in distinguishing between normal and abnormal movements, a receiver operating characteristic (ROC) curve will be plotted out by applying a grid of possible configurations of the two thresholds. Then, the area under the curve (AUC) is used to evaluate the method. If AUC is equal to one, it means that the anomaly can be perfectly separated from the normal movement.

In addition, the whole evaluation process is repeated three times with three different sources of input features (i.e., *Kinect Alone*, *Kinect+IMU*, *Marker-based*) to observe the effect of motion capture accuracy improvement in an anomaly detection task.

#### Experiment on post-stroke subjects

Normal movements from all 12 healthy subjects are used to build the model. Then, the model is used to process the movement from post-stroke subjects to get sequences of extracted phase values and anomaly scores.

### Parameter configuration

For the phase learning [23], the neural network is modified to have 5 hidden layers with 30 hidden nodes in each layer. The window size for phase smoothing is 7. The standard length of time warping and PCA is *n* = 50.

### Hidden Markov model as a baseline

The quantitative evaluation on simulated anomaly has been repeated on a Hidden Markov Model as a baseline for comparison. Hidden Markov Model (HMM) is a common unsupervised learning tool that can be used to model time-series data for anomaly detection. It can learn a probabilistic model from a set of sequences of normal movements. Then, the learned model can be used to estimate the probability of a newly observed sequence with a short observation window. Any output probability or score under a threshold is interpreted as detected anomaly.

For this experiment, HMM with Gaussian mixture emissions is chosen as it can model multi-dimensional data with continuous value. Each sample fed to the model has 11 dimensions. They are the normalized pose features after the AZEQ projection step. The model is configured to have 10 hidden states. Each probability density function to approximate the emission contains 5 Gaussian kernels with unrestricted covariance matrices. We allow the training process to run for a maximum of 1,000 iterations which takes around 13–52 min per model on Intel Core i7-6850K CPU. Each test sequence is a sliding window of 30 frames (1 s) with the stride of 1 frame. This implementation is done using *hmmlearn* Python library [36].

## Results and discussions

### Receiver operating characteristic (ROC) curve

**Fig. 8:**
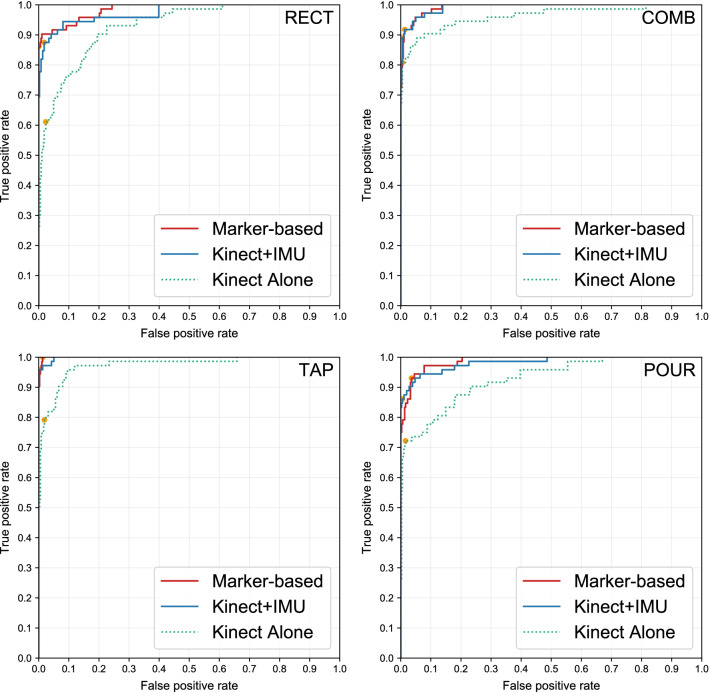
Receiver Operating Characteristic (ROC) curve from using the proposed method on the 4 exercises retrieved from the 6-fold cross-validation on 12 subjects. By enhancing the accuracy of the Kinect skeleton tracking with our motion capture method (*Kinect+IMU*), the accuracy of the anomaly detection task is also improved to the level that is comparable to the Marker-based motion capture system. Each orange point marks the point on the curve where the F1-score is maximized

Twelve ROC curves that are shown in Fig. 8 summarize the overall result. Our sensing system (*Kinect+IMU*) can achieve ROC curves that pass through the region with under 10% false positive rate and over 90% true positive rate (The grid on the top-left corner). This trend is observed in all four movements. Our procedure reaches a near-perfect degree of separation with the area under the curve (AUC) of 0.9924 and 0.9985 for COMB and TAP movements respectively. Nevertheless, a slight performance drop is found in the RECT and POUR movements with AUC of 0.9753 and 0.9827 respectively. For each movement, a pair of thresholds for anomaly detection is selected based on the optimum spot on the ROC curve with the highest F1-score. These thresholds can be seen on the plots of anomaly score sequence as horizontal gray dashed lines and the reported accuracies are generated from these optimum points. The details of those AUC and the optimal points are in Table 1.

**Table 1:** Evaluation result from the proposed method. Area under the curve (AUC) and accuracy at optimal points

Exercise	Feature source	AUC	Optimal point accuracy*		
			Precision	Recall	F-1 score
RECT	Kinect alone	0.9299	0.8302	0.6111	0.7040
	Kinect + IMU	0.9753	0.9000	0.8750	0.8873
	Marker-based	0.9851	1.0000	0.8611	0.9254
COMB	Kinect alone	0.9624	0.9672	0.8082	0.8806
	Kinect + IMU	0.9924	0.9702	0.8904	0.9286
	Marker-based	0.9928	0.9306	0.9178	0.9241
TAP	Kinect alone	0.9733	0.8906	0.7917	0.8382
	Kinect + IMU	0.9985	1.0000	0.9583	0.9787
	Marker-based	0.9994	0.9351	1.0000	0.9664
POUR	Kinect alone	0.9282	0.8966	0.7222	0.8000
	Kinect + IMU	0.9827	0.9688	0.8611	0.9118
	Marker-based	0.9882	0.8590	0.9306	0.8933

*The optimal point is the point where the F-1 score is maximized (the orange points in Fig. 8)

As illustrated in Fig. 8, the performance trends observed from three different motion capture systems are very similar. The motion sequence extracted from the Kinect SDK alone has the worst performance. The *Kinect+IMU* system improved the ROC curve to closely match the results from the Marker-based motion capture system despite the fact that the *Kinect+IMU* system is much more compact and much more affordable. The combination of the proposed method and the *Kinect+IMU* setup is the most suitable to be deployed for home-based rehabilitation.

**Fig. 9:**
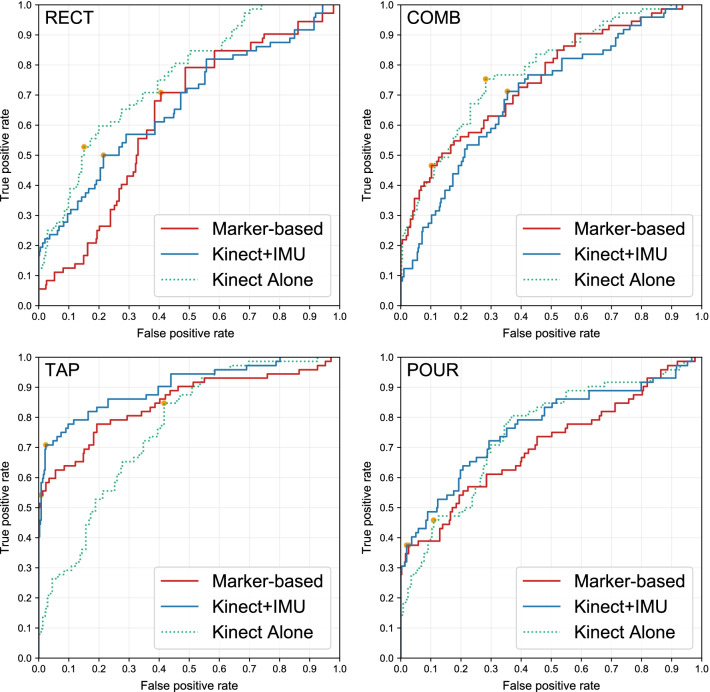
Receiver operating characteristic (ROC) curve from using the Hidden Markov Model method on 4 exercises retrieved from the 6-fold cross-validation on 12 subjects. Each orange point marks the point on the curve where the F1-score is maximized

In comparison to the proposed method, results from the HMM method (Fig. 9 and Table 2) show that HMM is not robust enough for this application. It produces an excellent AUC of 0.9019 for TAP movement but the value drops below 0.8 for the rest of the exercises. Nevertheless, the HMM baseline with an average AUC of 0.7520 performs worse than the proposed method with an average AUC of 0.9757.

**Table 2:** Evaluation result from the Hidden Markov Model method. Area under the curve (AUC) and accuracy at optimal points

Exercise	Feature source	AUC	Optimal point accuracy*		
			Precision	Recall	F-1 score
RECT	Kinect alone	0.7575	0.3958	0.5278	0.4524
	Kinect + IMU	0.6672	0.3025	0.5000	0.3770
	Marker-based	0.6281	0.2476	0.7083	0.3669
COMB	Kinect alone	0.7820	0.3438	0.7534	0.4721
	Kinect + IMU	0.7040	0.2781	0.7123	0.4000
	Marker-based	0.7506	0.4722	0.4658	0.4690
TAP	Kinect alone	0.7605	0.2798	0.8472	0.4207
	Kinect + IMU	0.9019	0.8500	0.7083	0.7727
	Marker-based	0.8491	0.9286	0.5417	0.6842
POUR	Kinect alone	0.7497	0.4459	0.4583	0.4521
	Kinect + IMU	0.7675	0.7941	0.3750	0.5094
	Marker-based	0.7057	0.7714	0.3750	0.5047

*The optimal point is the point where the F-1 score is maximized (the orange points in Fig. 9)

### Size of dimensionality bottleneck in reconstruction

To create a reconstruction process using principal component analysis, the minimum number of dimensions needed to create the information bottlenecks to preserve 95% of the variance in the training data in the three sensing systems are largely different. On average, to explain 95% of the variance in the training data, *Kinect Alone*, *Kinect+IMU*, and *Marker-based* need 36.7%, 16.4%, and 9.0% of the original number of dimensions respectively. These numbers reflect the difference in data quality extracted from those sources.

With the Kinect SDK alone, it needs many more dimensions at the bottleneck because of its noisier skeleton tracking. Especially for wrist-related and forearm-related features, the wrist-worn IMU sensors have considerably enhanced the tracking quality of the forearms and resulted in the required number of dimensions to decrease two to five times.

Still, the smallest number of dimensions at the PCA bottleneck always comes from the marker-based motion capture system regardless of the feature channels because of its highest accuracy.

### Computational performance and bottleneck

The training for the phase extraction network with around 100,000 pairs of training samples takes under 10 min on an Nvidia GTX 1080 Ti GPU to optimize for 10,000 iterations. The inference process using the trained network to calculate an instantaneous phase takes less than 1 ms on a single CPU core (Intel Core i7-3370).

Throughout the entire pipeline, the slowest part is in the motion capture step of *Kinect+IMU* which takes about 1.5 s per frame on a CPU (Intel Core i7-3370) due to a complex numerical optimization method reported in our previous work [33]. However, feature extractions from *Kinect Alone* and *Marker-based* systems can be done instantaneously. Other than the bottleneck in the motion capture step of *Kinect+IMU*, the rest of the calculations are mostly linear such as linear interpolation and matrix multiplication which can be done instantaneously on a modern CPU.

### Examples of the results

**Fig. 10:**
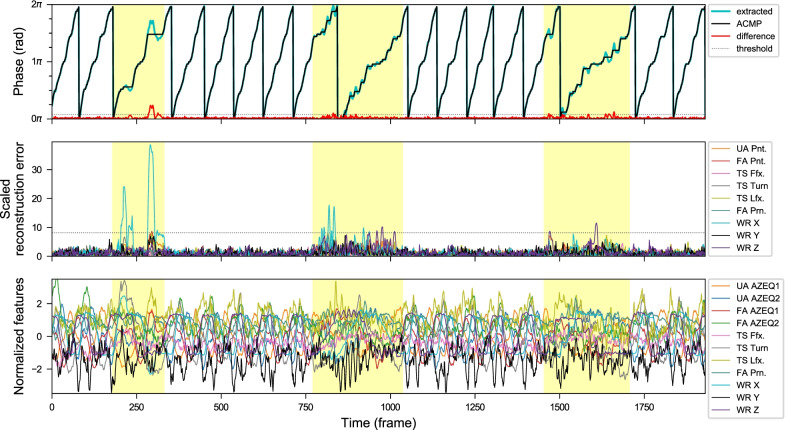
Anomaly detection results from sequences of RECT exercises with three simulated anomaly events recorded by *Kinect+IMU* system. These anomalous areas (highlighted in yellow) are manually labeled. Top: The extracted phase (without smoothing), and the approximately closest monotonically progressing (ACMP) phase sequence from the CDTW algorithm. The difference between them (red) is the anomaly scores from the phase fluctuation. Middle: The normalized reconstruction errors from different kinematic feature channels in different colors. Bottom: The normalized features used for the phase extraction

**Fig. 11:**
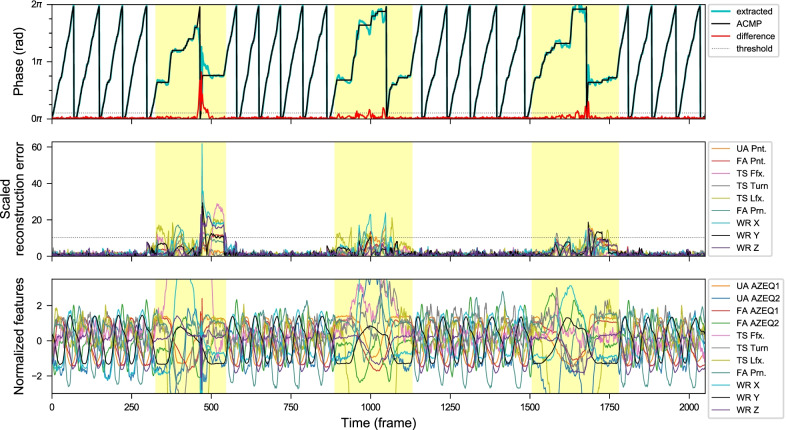
Anomaly detection results from sequences of COMB exercises with three simulated anomaly events recorded by *Kinect+IMU* system. These anomalous areas (highlighted in yellow) are manually labeled. Top: The extracted phase (without smoothing), and the approximately closest monotonically progressing (ACMP) phase sequence from the CDTW algorithm. The difference between them (red) is the anomaly scores from the phase fluctuation. Middle: The normalized reconstruction errors from different kinematic feature channels in different colors. Bottom: The normalized features used for the phase extraction

**Fig. 12:**
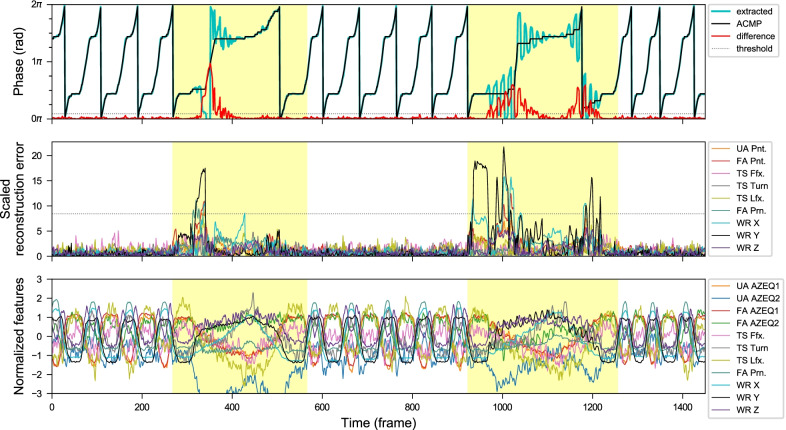
Anomaly plots from a POUR exercise sequence with high phase fluctuation (from *Kinect+IMU* system). The subject was asked to add zigzag and jerky movements to her trajectory in the first and the second highlighted section respectively. Notice that the proposed CDTW algorithm is able to retrieve the approximately closest monotonically progressing (ACMP) phase sequence for a reasonable period segmentation despite the considerable fluctuation in the original extracted phase sequence

**Fig. 13:**
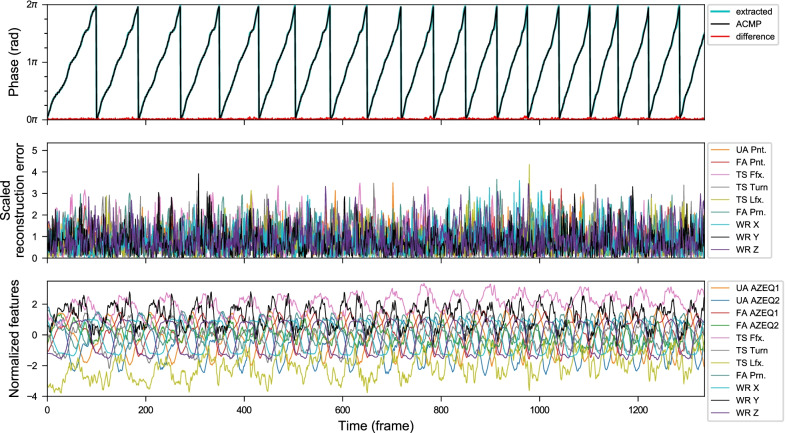
Anomaly plots from a RECT exercise sequence (from *Kinect + IMU* system) by a post-stroke subject with Fugl–Meyer UE motor subscore of 40 out of 42. The exercise is done with ease resulting in a clean phase progression and low reconstruction error. Notice that his pace is gradually faster over time

**Fig. 14:**
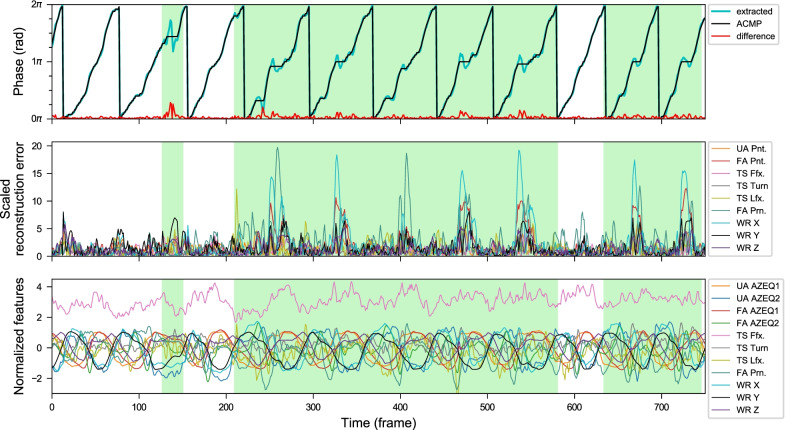
Anomaly plots from a COMB exercise sequence by a post-stroke subject (from *Kinect + IMU* system). The first highlighted segment is a short pause at the midpoint between the table and the head which is an unusual pausing point. For the second and the third highlighted segments, the high reconstruction errors and the phase fluctuations are caused by an abnormal path of movement from the back of the head to the table. She moves her hand to the space in front of her belly first before moving to the table. However, in some repetitions (non-highlighted areas), she did it normally without receiving any external feedback

**Fig. 15:**
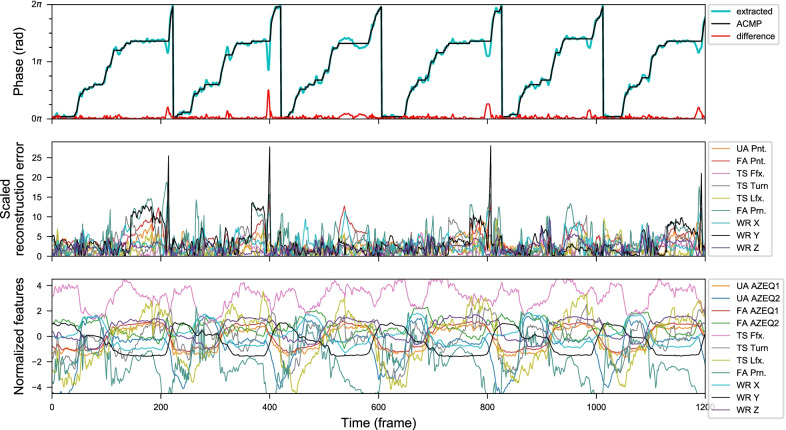
Anomaly plots from a TAP exercise sequence by a post-stroke subject (from *Kinect + IMU* system). Her arm constantly shakes whenever the hand is lifted against gravity and has no anchor point to push against. This shaking pulls the wrist position out from the proper trajectory sometimes. In addition, her forearm pronation keeps rolling back and forth throughout the exercise resulting in high reconstruction errors (FA Prn., dark cyan)

**Fig. 16:**
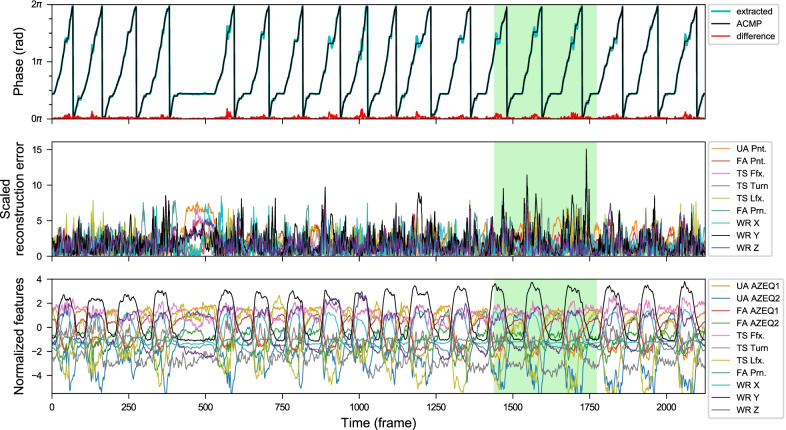
Anomaly plots from a POUR exercise sequence by a post-stroke subject (from *Kinect + IMU* system). He cannot extend his wrist to stay in a natural resting position. It causes his hand to always hang underneath the wrist level when he lifts the forearm. He has to compensate by lifting the wrist to about 10–15 cm higher than healthy subjects’ average. When the lift is too high, it is shown in the wrist height reconstruction error (WR Y, black). Phase fluctuations around the phase range of 1.0$\pi$ to 1.6$\pi$ that happen in most of his repetitions are caused by a small dip when the hand is above the bucket. By observation, a normal healthy subject usually lifts their hands just high enough to pour into the bucket without the dipping for the hand height adjustment. The long pause around the 500th frame is the moment when the cup is slipping out of his palm and he takes some time to adjust it

In the first experiment, some detection of simulated anomalies is illustrated in Figs. 10, 11 and 12. In the second experiment on post-stroke subjects, some results are shown in Figs. 13, 14, 15, and 16. The collected movements contain variations in the movement speed and anomalies. The details are explained in the captions.

### How will it help a therapist in exercise monitoring?

By skimming through the proposed compact representation of exercise movement, a therapist can easily determine the boundary of each repetition from the phase plot. The duration of phase sequence can also give an idea of how challenging the exercise is to a patient through how often the patient takes a break and how fast the phase progresses.

Furthermore, instead of inspecting a complex and high-dimensional waveform from all the features, the therapist can quickly identify an anomaly from the relatively high reconstruction errors in some feature channels.

With the highlighted key areas to take note from the phase and reconstruction error plots, the therapist can efficiently select specific sections in the exercise to review the video record and provide feedback to the patient.

## Conclusion

This study proposes a data-driven method to model repetitive movements with a compact representation. It allows a therapist to review long hours of rehabilitation exercises more effectively. Instead of looking through the whole exercise session, the therapist’s attention is directed to the section with high anomaly scores. The therapist’s expertise is then required to analyze the highlighted key areas and generate insightful feedback. This cooperation between the clinician and the machine could improve the quality of feedback generation and make telerehabilitation more scalable.

## Data Availability

The extracted features from this data collection are publicly available in a data repository [37].
